# Developing an Appropriate Digital Hearing Aid for Low-Resource Countries: A Case Study

**DOI:** 10.1155/2013/549486

**Published:** 2013-05-28

**Authors:** P. Israsena, S. Isaradisaikul, A. Noymai, S. Boonyanukul, A. Hemakom, C. Chinnarat, N. Navacharoen, S. Lekagul

**Affiliations:** ^1^National Electronics and Computer Technology Center (NECTEC), 112 Thailand Science Park, Pathumthani 12120, Thailand; ^2^Department of Otolaryngology, Faculty of Medicine, Chiang Mai University, Chaing Mai 50200, Thailand; ^3^The Rural ENT Foundation, RCOT, Soi Soonwichai, Huaykwang, Bangkok 10320, Thailand

## Abstract

This paper reviews the development process and discusses the key findings which resulted from our multidisciplinary research team's effort to develop an alternative digital hearing suitable for low-resource countries such as Thailand. A cost-effective, fully programmable digital hearing aid, with its specifications benchmarking against WHO's recommendations, was systematically designed, engineered, and tested. Clinically it had undergone a full clinical trial that employed the outcome measurement protocol adopted from the APHAB, the first time implemented in Thai language. Results indicated that using the hearing aid improves user's satisfaction in terms of ease of communication, background noises, and reverberation, with clear benefit after 3 and 6 months, confirming its efficacy. In terms of engineering, the hearing aid also proved to be robust, passing all the designated tests. As the technology has successfully been transferred to a local company for the production phase, we also discuss other challenges that may arise before the device can be introduced into the market.

## 1. Introduction

The World Health Organization (WHO) has estimated in 2005 that 278 million of the world population may have hearing difficulties [1]. In Thailand, data from the last survey by the National Statistical Office in 2007 indicates that there were 384,992 hearing impaired persons in Thailand [2]. Moreover, according to a national health survey during 2008-2009 [3], 28% of the elderly were found to have hearing difficulties. The situation will get worse as the country's demography is shifting towards an aging society. For those with hearing impairment who can benefit from wearing hearing aids, the use of such devices has not been widespread. Even in the USA, less than 25% of those who could benefit from using the hearing aids actually own them. The situation is worse in developing countries such as Thailand [4]. Historically, this was partly due to the analog technology that failed to provide the owners with satisfying results. Increasingly, however, modern hearing aids employ digital technology that shows significant improvements in terms of performance [5]. Many advanced features that were not available in analog technology are now being realized using digital signal processing (DSP) technology. Unfortunately, these devices come with higher prices and become unaffordable for users to benefit, especially in the developing countries.

In principle, hearing aids are devices that amplify and customize incoming sound signals to meet specific requirements of each hearing impaired. For a digital hearing aid, the sound is picked up via its microphone(s) and converted into the digital form that is processed according to predefined DSP algorithms tailored for each patient's requirements. The modified signal is converted back to the analog form for the receiver to convert it into sound waves and send them directly into the ear. Through this process, precise amplification characteristics desired can be achieved. Today, there are various types of hearing aids (both analog and digital) on the market, from body-aid (or pocket-type) to BTE (behind-the-ear), ITE (in-the-ear), ITC (in-the-canal), or CIC (completely-in-the-canal). These readily available devices offer choices on both price and performance. It is believed, however, that there are still demands for alternative hearing aid devices that are not readily available on the market. This can particularly be true in developing countries, where desirable features such as cost-effectiveness, ease of use and maintenance, and fully digital processing capability are imperative.

This paper discusses the development of an indigenous digital hearing aid targeted for rural usage. The result is a fully digital hearing aid that is designed for those with moderate-to-severe hearing loss. It comes in the shape of a pocket-type to meet the specific requirements for ease of use and robustness against extreme conditions of rural usage. The form factor also allows us to have an appropriate battery system that helps address the issue of maintenance cost. The hearing aid was systematically designed, engineered, and tested acoustically, functionally, and clinically. The design has now successfully been licensed out for local production.

The paper is organized as follows: Section 2 discusses development methods. This includes design and engineering issues and acoustical, clinical, and functional test setups.  Section 3 reports the results. Further discussions are given in Section 4, including issues to be considered before the device can be market ready.  Section 5 concludes the work.

## 2. Methods

### 2.1. Design and Engineering

#### 2.1.1. General Specifications

As hearing aids can vary vastly in terms of specifications and prices, it is imperative that the target for this project be the appropriate one. As guidelines for hearing aids and services for developing countries, the World Health Organization (WHO) has published a report in 2004 [6]. Our approach was to develop a hearing aid that betters or at least meets the technical requirements as specified on the report. The relevant criteria were selected as listed in Table 1.

#### 2.1.2. Fitting Range

Another important issue is the fitting range. As the majority of the elderly with hearing impairment fall into the class of having moderate to severe hearing loss, this would be the target group. It is important to design this early as it affects greatly the amplification characteristics needed. 

#### 2.1.3. Engineering and Functions

**Figure 1:**
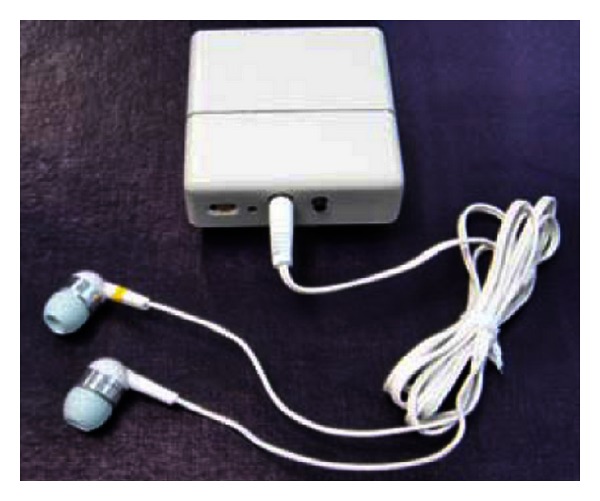
PDN-01B digital hearing aid developed.

**Figure 2:**
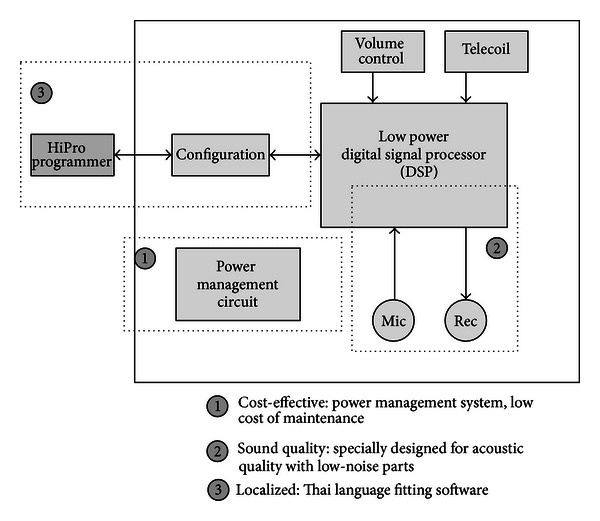
PDN-01B functional block diagram.

Our digital hearing aid, called the PDN-01B, is a pocket-type digital hearing aid. Even though it can be considered by some as old fashioned, the feedback that was gathered from users, especially those in the rural areas, suggested that there were still demands for a pocket-type device, which was considered more robust to extreme conditions and easier to operate. This is slightly different for the WHO recommendations, which prefer BTE to pocket-type. And we believe this was justified to pursue this pocket-type option based on our survey of actual needs of the local. The key was to provide a digital processing quality that would differentiate the hearing aid from the usual analog pocket aids, while offering the minimum cost of maintenance. The pocket-type device developed (Figure 1) is fully digital and is powered by one AAA battery. Its functional block diagram is given in Figure 2.

In engineering the device, we hoped to achieve the goals of cost-effectiveness, sound quality, and ease of use. The key components were the microphone, receiver, and the baseband processor (DSP). Although there are consumer grade parts available in the market, our design was based on using hearing-aid-specific parts, especially for these components. The main reasons for choosing these parts were to address the issues of current consumption that would affect its usage time before replacing the battery and the quality of the amplified acoustics. A hearing-aid-specific DSP is a low-voltage chip (1 Volt), which would offer superior current consumption over general DSP baseband counterparts (usually run on 3.5 Volt supply). Also, being a system on chip (meaning that all functions are included in one chip package) such as the hearing-aid-specific DSP usually implies lower power consumption when compared to equivalent systems built on discrete parts such as a DSP chip plus peripherals such as analog-to-digital converter (A/D) or digital-to-analog converter (D/A).

In terms of the sound quality, it is also imperative to have as little as possible noise in the system as it affects the quality of the amplified acoustics. Also, using a relatively flat response receiver will make the overall transfer function of the system more precise. For these reasons, a low-noise microphone and a flat response receiver, designed specially for hearing aids, were preferred over consumer-graded counter parts. Doing so has a small disadvantage in terms of the cost of the device, but we expect that saving from lower cost of maintenance will be higher than the additional cost of the device itself. Our third objective was ease of use. By designing the device as a pocket-type with minimal use of peripherals such as sockets and buttons, we aimed to make the device easy to use and maintain. The number of buttons was also reduced further by the fact that the hearing aid was designed to be fitted via computer rather than needed built-in trimmers. The Thai language fitting software also provided easy and precise fitting.

Based on the concept discussed, the PDN-01B digital hearing aid has key features as summarized in Table 2.

### 2.2. Acoustical Test

Amplification characteristics are arguably the most important features that will determine the usefulness of a hearing aid. Therefore, an approach to systematically and universally test and benchmark these devices via standardized tests is needed. Indeed, at present testing electroacoustics performance of hearing aids generally can be done to serve two purposes: (1) to verify that the instrument is functioning properly—according to the manufacturer's specifications and (2) to verify that the instrument is functioning appropriately—according to auditory needs of the wearer [7, 8]. In the first case, because the hearing aids are to be used by humans, the devices need to be objectively proven that they are safe to wear, and as much as possible they need to provide relevant functions such as amplification gains that will be useful for hearing impaired. Testing in the second case, on the other hand, is done after the device is fitted to the wearer, with real-ear measurement preformed to confirm that the electroacoustic characteristics during actual usage are as intended, for example, whether the gain measured in real ear is equal to the intended gain calculated.

For device development purposes, the measurement issues concerned here are with regard to the first purpose. Within the scope, the International Electrotechnical Commission (IEC) and American National Standards Institute (ANSI) are arguably the two world leading organizations for standardization of electroacoustic measurements in hearing aids. The most important sets of standards are the IEC-118 (IEC 60118) and ANSI-322 (ANSI S3.22) which cover most of the tests required. They are similar to a certain degree, and usually a hearing device certified for compliance with at least one of the standards should be sufficient.

Among these standards, IEC 60118-0 measurement of electroacoustical characteristics [7] and IEC 60118-7 measurement of the performance characteristics of hearing aids for quality inspection for delivery purposes [8] are the two most relevant measurements for consumer protection. As the name suggests, IEC 60118-0 provides basic details about measurements characteristics and set up schemes that are involved in hearing aid measurement. The standard defines the measurement of physical performance characteristics of air conduction hearing aids based on a free-field technique and measured with an ear simulator. It describes methods of measurement for evaluating electroacoustic characteristics of hearing aids. IEC 60118-7 is closely related to t 60118-0, but this part of IEC 60118 gives recommendations specifically for the measurement of the performance characteristics of air-conduction hearing aids of a particular model for production, supply, and delivery quality assurance purposes. The manufacturer will normally assign such nominal values upon delivery, and buyer can test and check its performance whether it conforms to manufacturer's claim. The standard provides information on how the hearing aids should be set up and what measurements are to be made. It is not intended to be used as a predictor for real-ear performance. IEC 60118-7 requires that the hearing aid needs to be measured in a sound-proof environment (usually in an anechoic box). The device is connected to a 2cc coupler that simulates simple ear volume. The hearing aid receives reference signal via a calibrated speaker and outputs amplified sound that is detected by the measurement microphone via the coupler. The result obtained is processed to provide corresponding electroacoustic data of the device. All necessary test parameters such as exact placements and angles between the devices and signal strength or characteristics are all specified in the standard.

**Figure 3:**
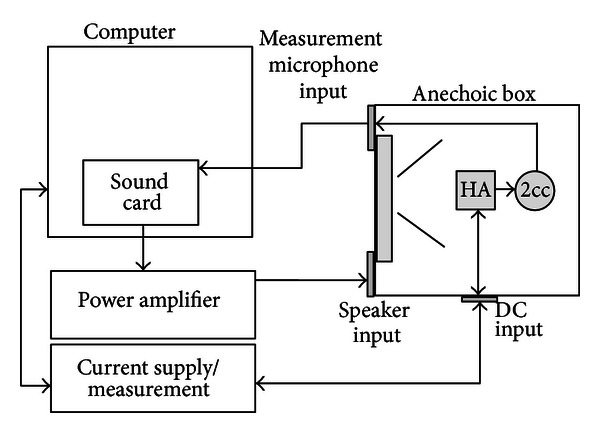
HA-TEST.

Our research team has set up our own electroacoustic testing facility that can support electroacoustic measurements according to the requirements set by the standards called the HA-TEST (Figure 3). It consists mainly of an anechoic box with 2cc coupler, computer with special sound card running professional sound measurement software, power amplifier, and a current supply/measurement device. To make sure about the accuracy of our test results, we compared initial test results of a sample device with ones obtained by having the same device tested by a certified laboratory abroad. The PDN-01B had gone through the electroacoustical tests following the IEC 60118-7 standard, with the measurement results as shown in Table 3. It can be seen that the fitting rage of the PDN-01B hearing aid suits those with moderate-to-severe impairment as intended.

### 2.3. Clinical Test

Apart from the needs to confirm its amplification characteristics, determined by electroacoustical measurements, another key test is to evaluate its real effects on wearer. This was done via a trial which aimed specifically to study users' satisfactions. The trial was carried out at Chiang Mai University, Thailand. It followed the prospective descriptive study approach, where descriptive statistic (both means and standard deviation) of the users' satisfaction was to be obtained from data analysis of handed-out questionnaires. The data was collected during the initial fitting, 1, 3, 6, and 12 months of followup. The candidates to this program needed to meet the criteria as stated in Table 4.

 The sample size was calculated for a single mean as follows:

(1)  n=4Zα2S2W2=35  cases, zα=type I error 5%=1.96, s=standard  deviation  of  EC  benefit=21,w=desired  total  width  of  mean  APHAB  score=±7=14.

The questionnaire used in this trial was the universally accepted Abbreviation Profile of Hearing Aid Benefit (APHAB) by Cox and Alexander [9]. It was translated into Thai language under permission, according to our understanding for the first time, based on the translation guidelines from Cox and the process of translation and adaptation of instrument of World Health Organization (WHO) [10, 11].

### 2.4. Long-Term Functional Tests

The objective of this one-year-trial phase was also for the aids to undergo scheduled acoustical and engineering tests, especially to evaluate their reliability. These tasks were done when each user visited the hospital for his/her routine followups (initial fitting, 1, 3, 6, and 12 months afterwards). They were (1) fitting and refitting, (2) acoustical measurements, and (3) component checking. The objective of the fitting and refitting was to check the ability of device to be refitted after the period of using. For each visit, the fitting data of each patient was recorded. The patient was either refitted with new set of parameters as determined appropriate by the audiologist or refitted with the same values if no adjustment was needed. Key engineering according to this part was to confirm that its ability to be refitted was intact. The second task was to check their acoustical performance. It was imperative that the hearing aid's amplification characteristics remained consistent. Although it was expected that some patients might need to adjust some fitting parameters during the followups, key acoustical parameters should remain within the intended rages. Here three parameters that can routinely be measured as part of IEC 60118-7 test that were the maximum output sound pressure level at input 90 dB SPL (Max OSPL90), the full-on gain (FOG), and lastly the total harmonic distortion (THD) were recorded. All measurements were performed onsite, using the FP35 Portable Hearing Aid Analysis available courtesy of the Rural ENT Foundation. 

## 3. Results

**Figure 4:**
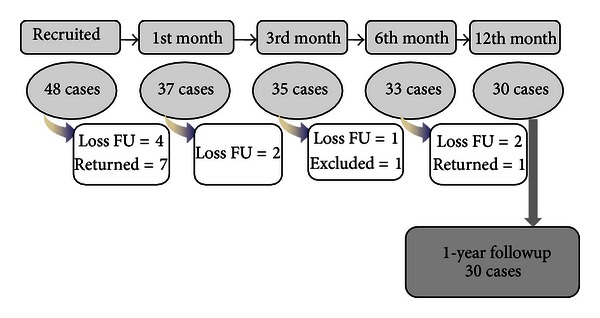
Trial overview (October 2009–July 2012).

The trial period was from October 2009 to July 2012. Overview of the completed trial is as illustrated in Figure 4.  Table 5 shows detail of the participants' characteristics.

### 3.1. User's Satisfaction

The result of users' satisfaction was determined via APHAB questionnaire. The APHAB questionnaire consists of 24 items. For each question, the hearing aid user is required to indicate at what level he/she agrees with the sentence; that is, A Always (99%), B almost always (87%), C generally (75%), D half-the-time (50%), E occasionally (25%), F seldom (12%), and G never (1%). The form was translated into Thai and used to evaluate the user's satisfaction of using the hearing aid in four main aspects that are

- ease of communication (the effort involved in communication under relatively easy listening condition, EC),
- reverberation (speech understanding in moderately reverberant rooms, RV),
- background noise (speech understanding in the presence of multitalker babble or other environment competing noise, BN),
- aversiveness of sounds (negative reaction to environmental sound, AV).

**Figure 5:**
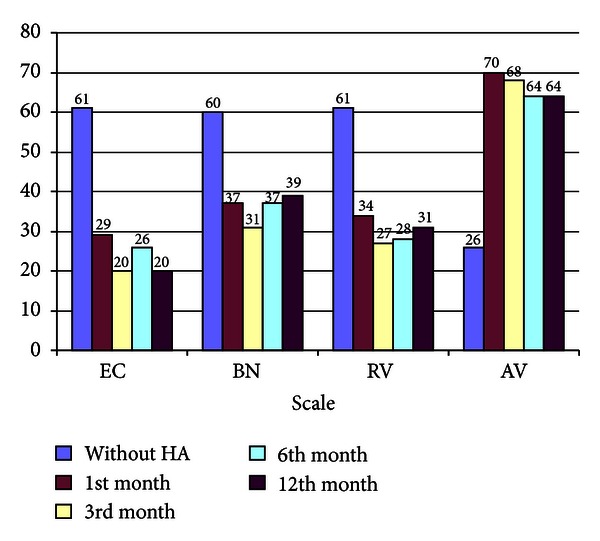
APHAB results.

The data was collected and compared for pre- and posthearing aid usage to determine its benefit. Results analyzed are as shown in Figure 5.

It can be seen that users were satisfied in terms of ease of communication (EC), reverberation (RV), and background noise (BN), with results found to be significant after 3 and 6 months. The relatively less-clear benefit in aversiveness of sounds (AV) is expected as amplification of unwanted noises is one of the most common problems faced when using a hearing aid. It is noted that similar results are obtained in Table 3 of [9] (although the studies were carried out on different groups of patients). The similarity in terms of the APHAB results makes these findings encouraging.

### 3.2. Acoustical and Functional Consistency

**Figure 6:**
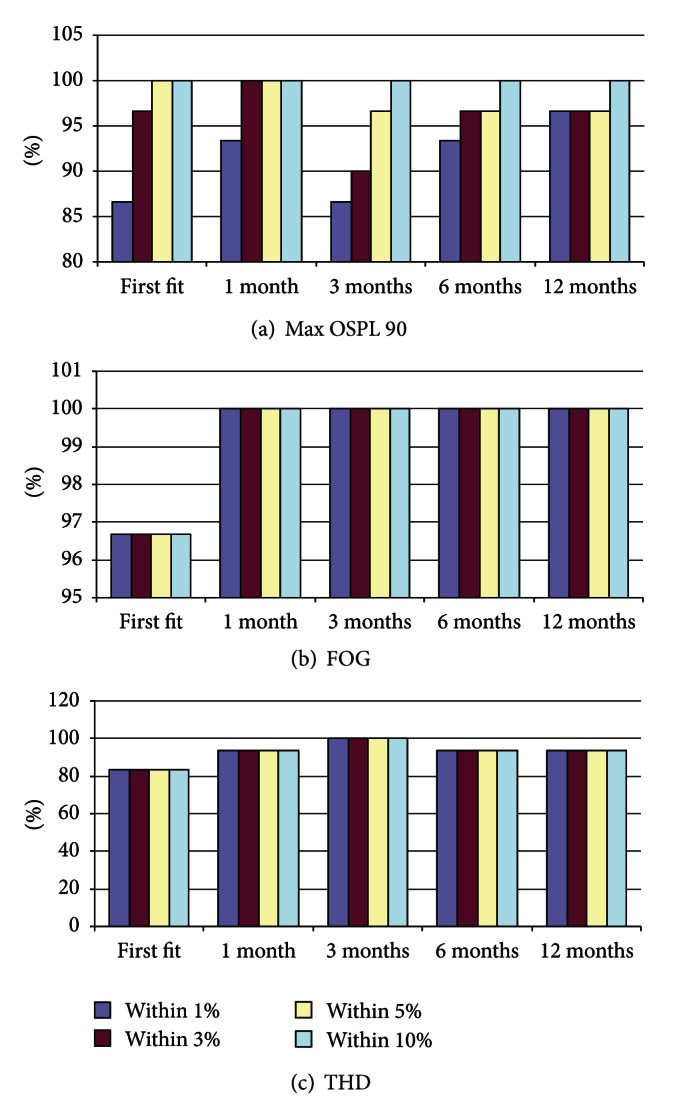
Consistency test results (acoustics).

Figure 6 summarizes the results from acoustical tests collected during the trail. Expectations are that, given the acoustical specification as in Table 3, Max OSPL90 should be less than 125 dB SPL (guarantee its safety), FOG should be at least 25 dB (confirm its amplification ability), and THD should be no more than 2% (indicate qualify of the sound) for all the follow-up measurements. Reading from the results, for Max OSLP90 all measurements are found to be within 10% of the target, and almost all are within 3%. All but one device passed the FOG test without any error. About 15% of the devices under trial were found to have THD of more than 2%, and if that happened, most of them would have the THD error of more than 10% from the target. It can be said that acoustical-wise the sample hearing aids performed reliably as expected. Some of the devices may have been shown to have distortion level higher than expected, but it is possible that this is due to the manufacturing process designed only for prototyping. The yield is expected to improve further during the mass production phase. Final engineering test concerns functional checking of major components. Those checked ones included the volume control, on/off switch, mode switch, and the light indicator. All of these have been shown to function well and none is broken.

## 4. Discussions

We have shown that it is possible to develop an indigenous hearing aid for low-resource countries. The key to the achievement is firstly to be precise about the requirements as this will affect greatly the final product. Developing the prototype requires mainly knowledge about embedded system and digital signal processing technology at the level that can be met by qualified experts from universities or research institutes. To build the device, components can be locally or globally sourced. The critical challenge is the fact that hearing aids are medical devices, and before they can be put to use, they need to pass necessary tests. Even more so they need to earn the trust of medical experts who will use the devices as part of the patient's rehabilitation program. What we have tried to achieve, hence, is to put the device through string of tests, functionally, acoustically, and clinically so that everyone can be certain about efficacy, safety, and reliability of the device. To achieve that, we had gone through some interesting stages from having to develop our own electroacoustics testing facility through designing our trial protocol involving a new APHAB questionnaire that was translated into Thai language. The satisfying results have let us convince a local manufacture to license out the product and prepare to scale up the manufacturing process to make it commercially available, our ultimate goal. To achieve that, we have been working on updates on some of the features such as styling and more importantly to make the battery system rechargeable that we hope to help save maintenance cost even further. The final version of the design should have the specifications as shown in Tables 6 and 7.

**Figure 7:**
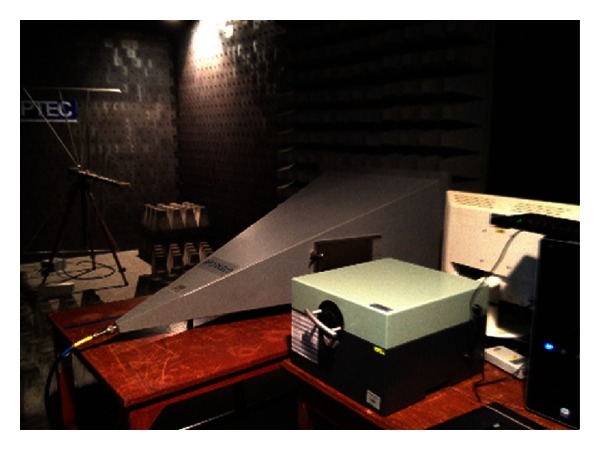
Under EMC test (IEC 60118-13).

In all, it should be noted that, from our experience, having a viable device only serves part of the solution. If this device is to make an impact, it needs to be competitive in the market and earning confidence of the experts. One way that may help towards achieving this could be to apply for certifications from international bodies, for example, the CE mark. To achieve this, the device needs to pass various standardized tests involving issues on safety, biocompatibility, or electromagnetic compatibility (EMC) (Figure 7), among others. CE also requires quality system (QS) on the manufacturing process, which can only be obtained by the manufacturer. Using the strategy of transferring the design to a local manufacturer with its own factory allows us to have all ingredients necessary to qualify for the CE mark, for which we are in the process of applying. Innovation on service delivery method would be another key ingredient in improving hearing rehabilitation services in low-resource countries such as Thailand, where experts such as audiologists are few and far between. We expect technologies such as teleaudiology [12] to be something that could be very useful. The area is another focus of our research.

## 5. Conclusion

This paper discusses the development of an indigenous digital hearing aid targeted for rural usage. The result is a fully digital hearing aid that is designed for those with moderate-to-severe hearing loss. It comes in the shape of a pocket-type to meet the requirements for ease of use and robustness against extreme conditions of rural usage. The form factor also allows for appropriate battery system that helps address the maintenance cost issue. The PDN-01B hearing aid was systematically designed and tested acoustically, functionally, and clinically. The design has now successfully been licensed out for local production.

## Figures and Tables

**Table 1:** Selected criteria from WHO guidelines for hearing aids and services for developing countries [6].

Technology related WHO's criteria on hearing aids	
(i) BTE is preferred, but body worn type may also be needed for low cost, ease of use, and availability of batteries, also in cases of rural areas where body worn is preferred	
(ii) Allowing ease of service, components available for five years; manufacturer provides sufficient detail	
(iii) HA should be powered by zinc air or secondary rechargeable cells; rechargeable types are preferred	
(iv) Operating in temperature 5–45 degree range, humidity 0–80%	
(v) Having means to reduce gain below 750 Hz	
(vi) Allowing at least 12 dB reduction at 250 Hz relative to 750 Hz	
(vii) Gain can be preset or adjusted by user	
(viii) At least 30 dB volume range, volume control clearly numbered	
(ix) Induction coil is an option but preferred	
(x) HA with AGC is preferred	
(xi) Manufacturer has ISO 9001 on quality management system	
(xii) HA should be designed such that risk of injury or discomfort to user is minimized	
(xiii) Manufacture should provide HA with at least few basic colors	
(xiv) The number of moving parts should be minimal	
(xv) DAI, FM, or other direct coupling input could be considered for educational settings but should not be considered priority if it jeopardizes the provision of hearing aids themselves	

**Table 2:** PDN-01B features.

Key features
(i) 100% digital
(ii) 2-channel WDRC
(iii) 5-band equalizer
(iv) 4 selectable memories
(v) Multimemory tone indicator
(vi) Low battery indicator
(vii) Powered by a single AAA battery
(viii) Telephone compatible via telecoil
(ix) Computer programmable via HiPro interface
(x) 4 adjustable optional trimmers
(xi) Standard 3.5 mm earphone

**Table 3:** PDN-01B electroacoustics measurements.

IEC 60118-7	Measured
HFA-OSPL90	105 dB
Max OSPL90	118 dB
HFA FOG 50 dB	53 dB
Low frequency cutoff	300 Hz
High frequency cutoff	7.7 kHz
THD at 70 dB	2.3%
Equivalent input noise	38 dB
Current drain	13 mA
Attack time	1.3 ms
Release time	382.9 ms
HFA-SPLITS	105.0 dB
STS	5.0 dB

**Table 4:** Inclusion criteria.

Criteria	
(i) More than 18 years old	
(ii) Able to read and understand the questionnaire, the manual, and the record form	
(iii) Has pure tone average air-conduction threshold at 500, 1000, and 2000 kHz between 40–90 dB in the better ear	
(iv) Suitable for hearing rehabilitation by using a hearing aid	
(v) Has never used a hearing aid or not satisfied with the current one	
(vi) Understand how to operate the hearing aid after training	
(vii) Can be back for followups after 1, 3, 6, and 12 months	

**Table 5:** Participants' characteristics.

Trial participants
(i) Total of 30 cases (9 males, 21 females)
(ii) Age average of 59.0 ± 13.7 years
(iii) Fitted ear (right 14, left 16)
(iv) Average air-conduction hearing of 65.1 ± 9.0 dB
(v) Speech reception threshold of 64.7 ± 12.6 dB
(vi) Speech discrimination score of 62.6 ± 25.5%
(vii) Average hours/day usage of 2.7 ± 0.7 hours
(viii) Average days/battery of 17.6 ± 9.2 days

**Table 6:** Specifications of the commercial-ready version.

Technology related WHO criteria	Commercial-ready version
BTE is preferred but body worn type may also be needed for low cost, ease of use, and availability of batteries, also in cases of rural areas where body worn is preferred	Body worn designed for rural usage
Allowing ease of service, components available for five years; manufacturer provides sufficient detail	Yes
HA should be powered by zinc air or secondary rechargeable cells; rechargeable types are preferred	Rechargeable
Operating in temperature 5–45 degree range, humidity 0–80%	Yes according to part datasheets
Having means to reduce gain below 750 Hz	Yes (low freq cutoff 380 Hz)
Allowing at least 12 dB reduction at 250 Hz relative to 750 Hz	Yes
Gain can be preset or adjusted by user	Programmable via computer
At least 30 dB volume range, volume control clearly numbered	Yes
Induction coil is an option but preferred	Induction coil provided
HA with AGC is preferred	AGC with WDRC
Manufacturer has ISO 9001 on quality management system	ISO 13485 (medical product QS)
HA should be designed such that risk of injury or discomfort to user is minimized	Yes
Manufacture should provide HA with at least few basic colors	Yes
The number of moving parts should be minimal	Yes
DAI, FM, or other direct coupling input could be considered for educational settings but should not be considered priority if jeopardizes the provision of hearing aids themselves	with Bluetooth input (option)

**Table 7:** Electroacoustics of the commercial-ready version.

Electroacoustics (IEC 60118-7)	Commercial-ready version
Max OSPL90 118 dB (±4 dB)	125 dB
OSPL90 @ 1 KHz 114 dB (±4 dB)	118 dB
Max FOG 45–55 dB (+5 dB)	71 dB
FOG @ 1 KHz 42 dB (+5 dB)	62 dB @ 50 dB
Basic frequency response 200–4500 Hz	380–4400 Hz
THD @ 70 dB SPL input 500 Hz < 5%, 800 Hz < 5%, 160 Hz < 2%	0.3%, 3.8%, 0.4%
Equivalent input noise level <25 dB SPL	25 dB
Battery current < 1 mA	11.8 mA (rechargeable)
